# Mutational heterogeneities in *STAT3* and clonal hematopoiesis-related genes in acquired pure red cell aplasia

**DOI:** 10.1007/s00277-025-06356-4

**Published:** 2025-04-09

**Authors:** Toru Kawakami, Fumihiro Kawakami, Shuji Matsuzawa, Taku Yamane, Yuga Mizuno, Ami Asakura, Daigo Higano, Shotaro Miyairi, Kaoko Sakai, Sayaka Nishina, Hitoshi Sakai, Yasushi Kubota, Yumiko Higuchi, Hideyuki Nakazawa, Fumihiro Ishida

**Affiliations:** 1 Department of Hematology and Clinical Oncology, Shinshu University School of Medicine, Matsumoto, Japan; 2 Central Laboratory Department, Shinshu University Hospital, Matsumoto, Japan; 3 Department of Biomedical Laboratory Sciences, Shinshu University School of Medicine, Matsumoto, Japan; 4 Division of Hematology, Respiratory Medicine and Oncology, Department of Internal Medicine, Faculty of Medicine, Saga University, Saga, Japan; 5 Department of Laboratory Medicine, Saga-Ken Medical Centre Koseikan, Saga, Japan; 6 Department of Health and Medical Sciences, Graduate School of Medicine, Shinshu University, Matsumoto, Japan

**Keywords:** STAT3, TET2, POT1, Clonal hematopoiesis, Pure red cell aplasia, Bone marrow failure

## Abstract

Dysregulation of T cell-mediated immunity is considered a major pathophysiological mechanism in acquired pure red cell aplasia (PRCA), including idiopathic PRCA, large granular lymphocytic leukemia-associated PRCA, and thymoma-associated PRCA. Although *STAT3* mutations are frequently detected in PRCA patients, the roles of other mutational profiles and their impact on clinical characteristics remain unclear. In this study, whole-exome sequencing and targeted sequencing using a custom-designed panel were performed on 53 PRCA patients. The most frequently mutated genes were *STAT3* (36%), *PCLO* (9%), *TET2* (9%), *NEB* (6%), *DNMT3A* (6%), and *POT1* (6%). Based on genetic profiles, patients were classified into three groups: those with *STAT3* variants (group S), those without *STAT3* variants but with variants in clonal hematopoiesis (CH)-related genes (group C), and those without variants in either *STAT3* or CH-related genes (group O). Patients in group C had a higher median age compared to group S, while group S exhibited milder anemia severity than group C. Additionally, *POT1* variants were associated with the idiopathic subtype of PRCA in females, often co-occurring with *STAT3* variants. Variants in CH-related genes and other genes, including *STAT3* and *POT1*, may play crucial roles in the pathophysiology of PRCA.

**Supplementary Information:**

The online version contains supplementary material available at 10.1007/s00277-025-06356-4.

## Introduction

Acquired pure red cell aplasia (PRCA) is an anemic disorder of bone marrow failure syndrome, defined by reticulocytopenia and a marked reduction or absence of erythroid progenitors in the bone marrow [1]. PRCA develops via T cell- or autoantibody-dependent immune mechanisms with a variety of underlying backgrounds. In particular, T cells play a crucial role in PRCA. T cells from patients have been shown to inhibit erythroid colony formation and/or erythroid cell lines [2–5], and an increase in CD8^+^ T cells and clonal T cells with characteristic immunophenotypes has been frequently recognized [6–9]. In addition, immunosuppressive therapies targeted at T cells such as cyclosporine A (CsA) have been quite effective and are the first choice for treating PRCA subtypes, including idiopathic PRCA, thymoma-associated PRCA, and large granular lymphocytic leukemia (LGLL)-associated PRCA [10–13].

Concerning the genetic background of PRCA, somatic *STAT3* mutations, particularly frequent in LGLL, have been detected in patients with various types of PRCA, including idiopathic, LGLL-associated and thymoma-associated PRCA [14, 15]. *STAT3* mutations are restricted to CD8-positive (CD8^+^) T cells [14]. Other studies have identified variants in several genes such as *KMT2D*, *KDM6A*, and *BCOR* [16–18]. However, many questions remain, particularly regarding the relationship between mutational profiles and the clinical characteristics of PRCA patients.

To determine the genetic profile and its relationship with clinical features, we performed whole-exome sequencing (WES) and targeted sequencing analyses on a large cohort of patients with PRCA, using an originally designed gene panel.

## Materials and methods

### Patients

PRCA patients were enrolled in this study, as were LGLL patients without PRCA and aplastic anemia (AA) for comparison. The diagnostic criteria for these diseases used in this study are summarized in Table S1 [19–22]. Clinical data, including age, underlying conditions, and laboratory data, were collected from medical records. Data on the therapeutic medications for patients with PRCA and their outcomes were obtained. The response criterion for PRCA [23] was also adopted.

This study was conducted in accordance with the Declaration of Helsinki and was approved by the Institutional Review Board of Shinshu University School of Medicine (approval number 723) and each participating center. Written informed consent was obtained from all the patients and healthy controls.

### WES

WES was performed using Ion AmpliSeq technology. DNA was extracted from CD4^+^ or CD8^+^ T cells of PRCA patients. The libraries were prepared using the Ion AmpliSeq Exome RDY Kit according to the protocol for preparing Ion AmpliSeq libraries (Thermo Fisher Scientific, Waltham, MA, USA). DNA concentrations in the libraries were measured using the Ion Library TaqMan Quantitation Kit (Thermo Fisher Scientific). The libraries were subjected to WES on Ion S5 according to the manufacturer’s standard protocol using the Ion 540 Chip Kit (Thermo Fisher Scientific). Data were analyzed using the Torrent Suite software program (v5.12.1; Thermo Fisher Scientific) and Ion Reporter software program (v5.12; Thermo Fisher Scientific). The variants were called using the workflow “AmpliSeq Exome single sample (Somatic).” The main variant calling settings were as follows: variant frequency filter, 0.02; base quality Q-value, ≥ 6.5; minimum coverage depth, 20; maximum strand bias, 0.9 (single nucleotide polymorphism (SNP)], 0.85 (INDEL). Variants that were considered SNPs or synonymous variants were eliminated. Variants with a variant allele frequency (VAF) of 20%− 40% from CD8^+^ T cells were compared with CD4^+^ T cells, and the variant characteristics of CD8^+^ cells were selected.

### Target sequencing

Target sequencing was performed using Ion AmpliSeq technology. Candidate genes were selected from the WES results, and genes related to clonal hematopoiesis (CH) or lymphoproliferative disorders were also included. Primers were designed to cover 97% of the coding sequences of candidate genes using the AmpliSeq Designer system (Thermo Fisher Scientific). The genes analyzed are summarized in Table S2. Libraries were prepared using the Ion AmpliSeq Library Kit Plus (Thermo Fisher Scientific) according to the manufacturer’s protocol. DNA concentrations in the libraries were measured using the Ion Library TaqMan Quantitation Kit (Thermo Fisher Scientific). The libraries were subjected to amplicon sequencing on the Ion GeneStudio S5 system according to the manufacturer’s standard protocol using the Ion 530 or 540 Chip Kit (Thermo Fisher Scientific). The data were analyzed using the Torrent Suite software program (v5.8.0; Thermo Fisher Scientific). The main variant calling settings were as follows: variant frequency filter, 0.01; base quality Q-value, ≥ 20; minimum coverage depth, 1000; and maximum strand bias, 0.95 (SNP), 0.9 (INDEL). The called variants were annotated using wANNOVAR (http://wannovar.wglab.org/index.php), and variants considered SNPs or synonymous variants were eliminated. Variants with VAFs ≥ 40% were not considered somatic mutations. Variants with VAFs ≤ 2% were excluded due to a high likelihood of error. However, *STAT3* variants with VAFs of 1–2% were included, as their reproducibility has been confirmed by previous reports. [14].

## Results

### Patient demographics

A total of 53 PRCA patients were included in the study. Peripheral blood samples were collected from all the patients. For the control groups, we included 21 LGLL patients without PRCA and those with aplastic anemia (AA). The PRCA subtypes were idiopathic (*n* = 11), LGLL-associated (*n* = 26), thymoma-associated (*n* = 10), autoimmune disease-associated (*n* = 3), and others (*n* = 3). The etiologies of AA were idiopathic (*n* = 9) and paroxysmal nocturnal hemoglobinuria (*n* = 1). The clinical characteristics of the patients are summarized in Table 1. The subtypes of LGLL with PRCA included CD8^+^TCRαβ (*n* = 17), CD4^+^TCRαβ (*n* = 1), TCRγδ (*n* = 5), and NK-LGLL (*n* = 3). The subtypes of LGLL without PRCA were CD8^+^TCRαβ (*n* = 19) and TCRγδ (*n* = 2).

**Table 1:** The clinical characteristics of patients

	PRCA							
	Whole	Idiopathic	Thimoma-associated	LGLL-associated	Autoimmune disease-associated	Others	T-LGLL without PRCA	AA
No. of patients	53	11	10	26	3	3	21	10
Age, median, y	65 (16–86)	72 (35–86)	64 (43- 85)	67 (16- 85)	63 (27- 67)	64 (49- 65)	64 (41- 86)	58 (27- 77)
Male, %	43	18	50	19	67	67	38	50
WBC, median, × 10^9^/L	5.3 (1.6–19.1)	4.3 (2.0–7.6)	5.0 (1.8- 8.7)	6.0 (1.6- 19.1)	5.7 (4.0- 6.0)	3.1 (2.9- 10.9)	4.4 (0.7–17.4)	2.3 (1.4- 3.3)
Neutrophils, median, × 10^9^/L	2.1 (0.3–13.1)	2.1 (0.9- 4.5)	3.5 (0.7- 5.7)	1.7 (0.3- 13.1)	2.9 (2.2- 3.4)	1.8 (1.5- 8.7)	0.8 (0–35.3)	0.9 (0.3- 1.9)
Hemoglobin, median, g/dL	67 (40–100)	53 (42- 78)	56 (40- 84)	73 (40- 100)	72 (67- 74)	69 (57- 70)	99 (69–160)	88 (49- 146)
Platelets, median, × 10^9^/L	303 (59–613)	303 (59- 613)	283 (213- 543)	299 (161- 584)	362 (208- 386)	314 (243- 319)	236 (5–437)	30 (11- 83)
Reticulocytes, median, × 10^9^/L	9.1 (1.7–46.6)	7.9 (3.1- 30.0)	6.2 (1.7- 3.2)	12.7 (2.1- 46.6)	7.0 (4.1- 8.3)	10.9 (9.2- 12.3)	59 (0–84)	57.5 (0.6- 78)
Follow-up duration, median, mo	62 (1–331)	49 (7- 255)	13 (2- 308)	92 (8- 331)	79 (39- 171)	76 (1- 152)	36 (0–308)	28 (0- 313)

*PRCA* pure red cell aplasia, *LGLL* large granular lymphocytic leukemia, *AA* aplastic anemia, *WBC* white blood cells

### WES of CD4^+^ T cells and CD8^+^ T cells in PRCA

The PRCA backgrounds analyzed with WES were as follows: idiopathic (*n* = 2), thymoma or thymic cancer (*n* = 5), and autoimmune diseases (*n* = 2). The median numbers of CD4^+^ and CD8^+^ T cells in the peripheral blood of the patients were 0.54 × 10^9^/L (0.44–0.68 × 10^9^/L) and 1.08 × 10^9^/L (0.29–2.25 × 10^9^/L), respectively. In this sequencing analysis, the median depth of coverage was 84x (range: 31–123) for CD4^+^ T cells and 136x (range: 114–205) for CD8^+^ T cells. The detected variants with VAFs of 20%− 40% are shown in Table S3 and Figure S1. The median number of mutated genes detected was 12 (range: 9–17) for CD4^+^ T cells and 11 (range: 1–17) for CD8^+^ T cells. None of the mutated genes were shared across samples. We included *HIPK4*, *MUC1*, and *SPAG5* as candidate mutated genes in CD8^+^ T cells and subjected them to target sequencing. We also added several mutated genes in samples derived from CD4^+^ and CD8^+^ T cells, including *TET2*, *HCFC1*, and *NHS*, for the panel.

### Landscape of mutations in PRCA

**Fig. 1:**
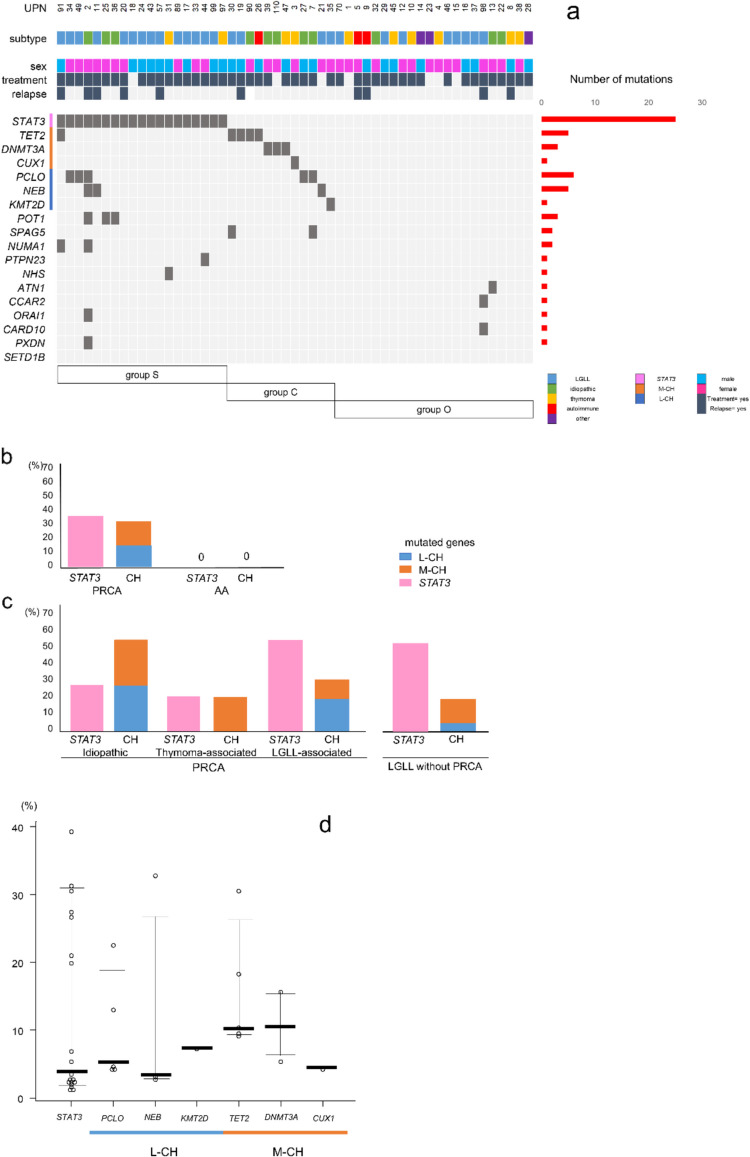
Mutational landscape of PRCA. **a** Oncoplot of PRCA. Each row represents a gene mutated in at least one case, while each column represents an individual patient. The backgrounds of PRCA are color-coded at the top. Clinical phenotypes are displayed above the heat map, and the bar graph on the right indicates the total number of mutations detected in each gene. Mutational subgroups (groups S, C, and O) are shown below the heat map. **b**, **c** Frequencies of CH-related genes and *STAT3* variants in PRCA and AA (**b**), and in relation to subtypes of PRCA and LGLL without PRCA (**c**). **d** Distributions of VAFs of *STAT3* and CH-related genes in PRCA. Bars indicate median values with first and third quartiles. PRCA, pure red cell aplasia; UPN, unique patient number; LGLL, large granular lymphocytic leukemia-associated PRCA; idiopathic, idiopathic PRCA; thymoma, thymoma-associated PRCA; autoimmune, autoimmune disease-associated PRCA; M-CH, myeloid clonal hematopoiesis-related genes; L-CH, lymphoid clonal hematopoiesis-related genes; AA, aplastic anemia; VAFs, variant allele frequencies

To gain further insight into the genetic profiles of patients with PRCA, we analyzed MNC-derived DNA from individuals with PRCA, T-LGLL, AA, and healthy controls by performing amplicon sequencing with a custom-designed panel. The median depth of coverage in this sequencing analysis was 3,368 × with a range of 1,665–5,454. *MUC17* and *IGFN1* were excluded from further analyses because of their high false-positive rates. The landscape of gene mutations in PRCA is shown in Fig. 1a. The identified mutations are summarized in Table S4. Thirty-three patients (62%) with PRCA exhibited at least one variant among the 50 genes included in the panel. Variants of eight genes were detected in multiple individuals. The mutated genes included *STAT3* (36%), *PCLO* (9%), *TET2* (9%), *NEB* (6%), *DNMT3A* (6%), and *POT1* (6%) (Figure S2). The most frequently mutated genes within each subtype were as follows: *PCLO* (27%), *POT1* (27%), and *STAT3* (27%) in idiopathic PRCA; *STAT3* (20%), *DNMT3A* (10%), and *CUX1* (10%) in thymoma-associated PRCA; and *STAT3* (54%) and *TET2* (12%) in LGLL-associated PRCA.

### Variants of CH-related genes and *STAT3* in PRCA and AA

**Fig. 2:**
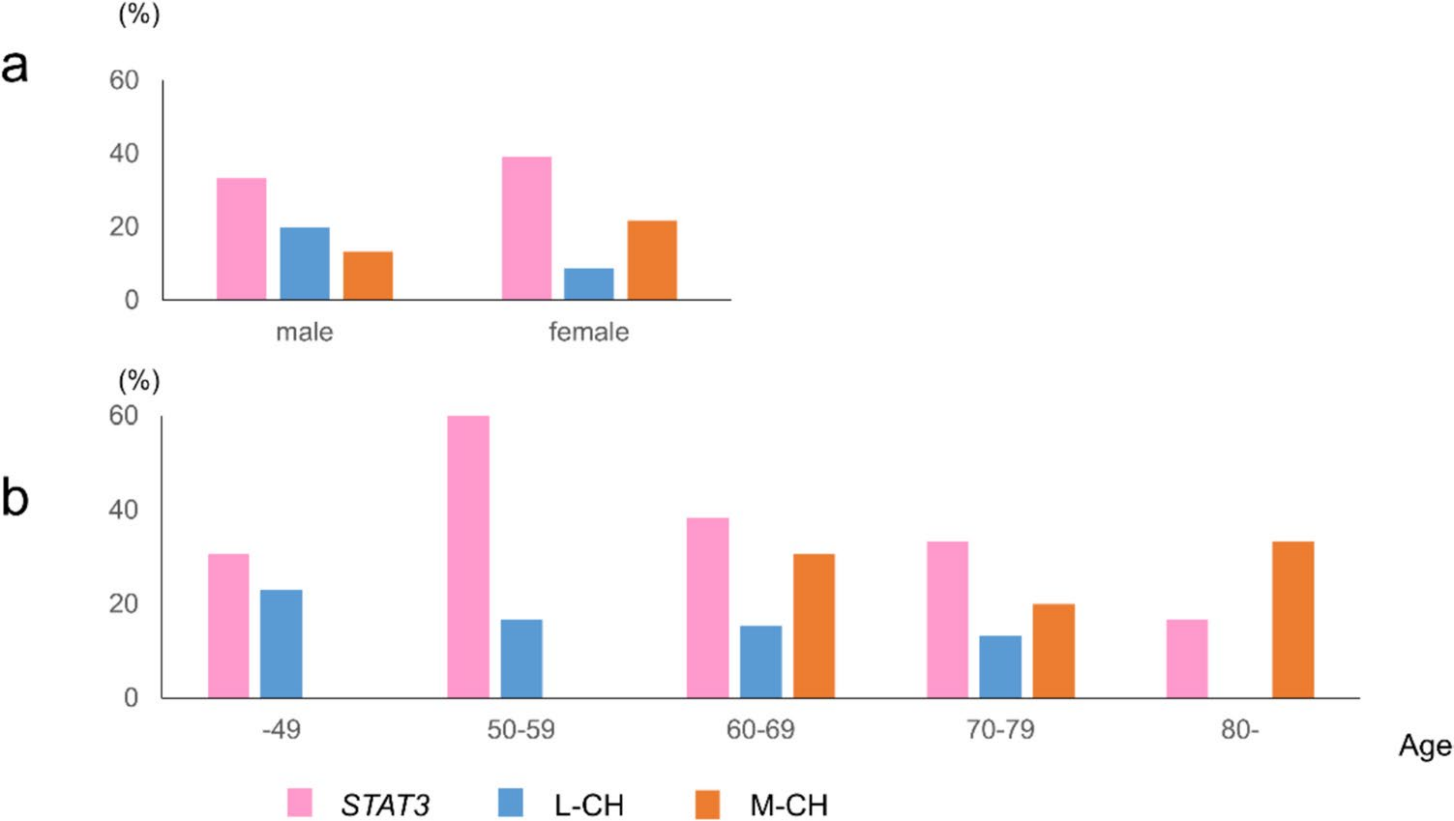
Frequencies of *STAT3* and clonal hematopoiesis-related genes variants. Each bar shows variant frequencies of *STAT3* and L-CH- and M-CH-related genes by sex (**a**) and ages (**b**). L-CH, lymphoid clonal hematopoiesis; M-CH, myeloid clonal hematopoiesis. The details of L-CH- and M-CH-related genes are described in the main text

We categorized *TET2*, *DNMT3A*, and *CUX1* as myeloid clonal hematopoiesis (M-CH)-related genes and *NEB*, *PCLO*, and *KMT2D* as lymphoid CH (L-CH)-related genes following Niroula et al. [24]. In PRCA patients, variants of L-CH- and M-CH-related genes were detected in 15% and 17% of cases, respectively. Of the 33 patients with at least one variant, 31 (94%) harbored variants in *STAT3*, L-CH-related genes, or M-CH-related genes. No patients with PRCA harbored variants in both L-CH- and M-CH-related genes (Fig. 1b), confirming the mutual exclusivity of these mutations. The frequencies of L-CH- and M-CH-related gene variants among PRCA subtypes, as well as the VAF of each CH-related gene, showed no significant differences (Fig. 1c, d). Variants observed in AA are summarized in Figure S3. No *STAT3* or CH-related gene variants were detected in AA patients. L-CH-related gene variants were more commonly found in younger patients, while M-CH-related gene variants were predominantly associated with age (Fig. 2a, b). None of the patients with variants in CH-related genes developed hematological malignancies, except for LGLL, during the median follow-up period of 5.1 years.

### Mutational subtypes in PRCA and their relationship with clinical findings

Based on mutational profiles, the following patterns were identified: *STAT3*-positive (group S, *n* = 19), *STAT3*-negative but positive for any CH-related genes (group C, *n* = 12), and negative for both *STAT3* and CH-related genes (group O, *n* = 22). Group S tended to have more LGLL-associated PRCA cases; however, there were no significant differences in the overall PRCA background. Group S included a subgroup with *POT1* or CH-related gene variants.

The gender distribution did not differ significantly among groups. However, the median age of patients in group C was significantly higher than that of group S (group C: 74.0 years vs. group S: 64.0 years, *p* = 0.016). Patients in group S had relatively higher hemoglobin levels compared to group C (group S: 71 g/L vs. group C: 63 g/L, *p* = 0.066) and significantly higher reticulocyte counts (group S: 16.2 × 10⁹/L vs. group C: 5.5 × 10⁹/L, *p* = 0.023). The percentage of patients receiving immunosuppressive therapies and their therapy responses did not differ significantly between groups (Table 2). However, patients with *STAT3* variants and those with multiple gene variants may exhibit a tendency toward refractoriness to CsA and a higher likelihood of recurrence after CsA compared to patients without *STAT3* variants and those without multiple gene variants, respectively (11/15 vs. 12/26, *p* = 0.063; 9/11 vs. 14/30, *p* = 0.13). Additionally, patients with multiple gene variants may also show a lower response rate to first-line immunosuppressive therapy (5/12 vs. 23/34, *p* = 0.17) (Supplemental Table S5, 6).

**Table 2:** Comparison among the mutational subgroups

	group S, *n* = 19	group C, *n* = 12	group O, *n* = 22	*P*
Age, median (range), y	64 (16–85)	74 (42–86)	63 (27–85)	* 0.016
Male, *n* (%)	9 (47)	6 (50)	8 (36)	0.68
WBC, median(range), × 10^9^/L	5.20 (1.78–19.13)	5.38(1.60–8.00)	5.52 (2.00–10.93)	0.78
Neutrophils, median (range), × 10^9^/L	1.49 (0.28–13.20)	3.13 (0.84–4.48)	2.30 (0.72–8.74)	0.056
Lymphocytes, median (range), × 10^9^/L	2.90 (0.05–9.86)	1.63 (0.42–5.60)	1.85 (0.68–5.52)	0.25
CD8^+^T cells, median (range), × 10^9^/L	1.15 (0–7.64)	0.52 (0.20–5.03)	1.08 (0.21–4.93)	0.39
Hemoglobin, median (range), g/L	71 (50–100)	63 (42–90)	63 (40–99)	0.061
Reticulocytes, median (range), × 10^9^/L	16.2 (2.1–46.6)	5.5 (1.7–16.8)	10.0 (2.6–34.6)	* 0.023
Platelets, median (range), × 10^9^/L	250 (161–613)	305 (144–511)	319 (59–584)	0.56
LD, median (range), IU/L	204 (147–350)	171 (139–273)	194 (137–405)	0.41
Erythroblasts in BM, median (range), %	1.6 (0.2–20.7)	0.8 (0–8.2)	0.5 (0–3.4)	0.46
Therapy				
CsA	15	9	17	1
CY	12	3	5	0.66
Others	5	3	8	0.62
Response to 1-st line therapy	10/18	7/10	11/18	0.80
Relapse after immunosuppressive therapies	5/18	1/10	4/18	0.60
Refractory to CsA	11/15	4/9	8/17	0.17

^*^Significantly different

*group S* *STAT3*-positive, *group C* *STAT3*-negative and positive for any CH-related genes

*group O* negative for both *STAT3* and CH-related genes, *PRCA* pure red cell aplasia, *CsA* cyclosporine, *CY* cyclophosphamide

*POT1*, a gene associated with telomere maintenance, was mutated in 3 of the 84 patients with hematological disorders (Table S7). All three patients were female, had idiopathic PRCA, tested positive for T-cell clonality and *STAT3* mutations, and were relatively young. Two were in their fourth decade of life. Variants in *POT1* were located near the N-terminus, with VAFs ranging between 2 and 6% (Figure S4). Among PRCA patients with *STAT3* variants, *POT1* mutations were exclusively observed in those with low *STAT3* VAFs (Figure S5). These findings suggest that *POT1*-mutated PRCA represents a novel subgroup of idiopathic PRCA with distinct characteristics.

### Somatic and germline *TET2* variants in PRCA

Somatic *TET2* variants were identified in five patients with PRCA, with a median VAF of 9.6% (range: 1%–30.5%). Three variants (R1516X, C1193Y, and I1873 T) have been reported in COSMIC (v101, released 2024-Nov- 19) as being associated with myeloid malignancies. However, PRCA patients with *TET2* mutations showed no dysplastic features in bone marrow cells, no increase in blasts, and no abnormal karyotypes.

Targeted sequencing identified shared variants between familial cases (UPN 11 and 20), limited to *STAT3* Y640 F and *TET2* N813S, with *TET2* variant VAFs ≥ 40%. Focusing on *TET2* variants with VAFs ≥ 40% revealed 4 additional patients were identified. The *TET2* variants are summarized in Figure S6 and Table S8. Sanger sequencing of DNA from the buccal mucosa of UPN 9, 11 and 35, confirmed the corresponding *TET2* variants, strongly suggesting germline *TET2* mutations in these cases. UPN 9 and 35 had no family history of cytopenia or hematological malignancies. Germline *TET2* variants (F387Y, N813S, and R881 W) have not been previously reported in COSMIC or other known germline variant databases [25–27]. Of the patients with *TET2* variants, 25% (UPN 11, 20, and 91) had *STAT3* variant commutations.

### Variants in T-LGLL with or without PRCA and *STAT3* variants

In LGLL, *STAT3* mutations are associated with PRCA [28], and *STAT3* mutations are frequently found in other subtypes of PRCA as well [14]. When comparing mutational profiles beyond *STAT3* in T-LGLL with or without PRCA (Table S9), the genes most frequently mutated in LGLL without PRCA were *STAT3* (48%) and *TET2* (10%). In the CD8^+^TCRαβ subtype, patients with PRCA exhibited significantly lower VAFs for *STAT3* mutations than those without PRCA (2.3% vs. 18.9%, *p* = 0.042). Patients positive for *STAT3* mutations without PRCA were of very old age (over 80 years) or possessed *STAT3* mutations with high VAFs (Figure S7).

## Discussion

In this study, we provided a comprehensive mutational profile of PRCA. In addition to *STAT3*, CH-related genes were frequently identified, and variants of L-CH-related genes were detected as often as those of M-CH-related genes in PRCA. We identified three mutational subgroups in PRCA: *STAT3*-positive (group S), *STAT3*-negative but positive for any CH-related genes (group C), and negative for both *STAT3* and CH-related genes (group O). The age of onset and severity of anemia varied across these groups. Group S predominantly consisted of LGLL-associated PRCA, with abnormalities in cellular immunity likely playing a significant role in its pathophysiology. Group C included individuals with a later age of onset, where the accumulation of mutations in hematopoietic stem cells might contribute to anemia development.

The mutational profile and clinical characteristics of group O remain unclear, underscoring the need for more comprehensive genetic analyses. These findings suggest that the heterogeneity of PRCA may be partially attributed to differences in genetic mutation profiles.

Variants of L-CH-related genes were much less frequent than those of M-CH-related genes in the general population without hematological abnormalities [24]. *NEB* and *PCLO* variants were infrequent in T-cell lymphomas, including T-LGLL [29, 30]. In this study, *TET2* and *DNMT3A* were classified as M-CH-related genes according to Niroula et al. [24]. Mutations in these genes have also been shown to be associated with lymphoid malignancies [31], which suggests that *TET2* and *DNMT3A* may be classified as L-CH as well. Consequently, L-CH-related gene variants might be more closely related to the pathophysiology of PRCA than those of M-CH-related genes.

The lower VAFs of *STAT3* mutations in LGLL-associated PRCA than those LGLL without PRCA, which was consistent with a previous report [32], and frequent clonal T cells negative for *STAT3* mutations in other subtypes of PRCA imply that *STAT3*-mutated T cells might not directly affect impaired erythropoiesis of T cell-mediated PRCA. Rather, complex and heterogeneous T cells and other cells with various backgrounds, including variants of lymphoid and/or myeloid CH-related genes, in addition to *STAT3,* contribute to the pathogenesis of PRCA. PRCA patients with *STAT3* mutations could be classified into two groups, one is *STAT3* high VAFs and the other is *STAT3* low VAFs (Figure S5 and S7). Patients with *STAT3* variants of high VAFs had relatively fewer mutations of CH-related genes and VAFs of *STAT3* seemed to increase with an age-dependent manner. On the other hand, patients with *STAT3* variants of low VAFs had more mutations of CH-related genes and/or *POT1* with no relationship with age. Patients in these two groups may develop PRCA through different mechanisms.

The subjects who were positive for L-CH-related variants were at a higher risk of developing mature B cell malignancies, including chronic lymphocytic leukemia as described in a previous report [24], than those without L-CH-related variants, although the risks of T cell disorders have not been well studied. We included only three L-CH-related genes in our target gene panel, which excluded highly mutated genes, such as *DUSP22*, *FAT1* or *ATM* [24]. Patients may be found to possess other gene variants when analyzed using more comprehensive methods. Further research is needed to determine the relationships between variants of *STAT3* and CH-related genes and cell lineages and their developmental processes.

Among the M-CH-related genes, *TET2* variants were most frequently detected in PRCA, whereas *DNMT3A* was the most frequently mutated gene in AA [33] and age-related CH [34]. Frequent *TET2* variants with a high VAF among various subtypes of PRCA regardless of age suggest important roles of TET2 in the pathogenesis of PRCA. *TET2* variants in PRCA were also reported by Fujishima et al. with a frequency of 8% (3/38) [18]. The five germline *TET2* variants detected in this study might contribute to the clinicopathological features of PRCA, based on the extremely low frequencies in public databases. It is also assumed that those mutations have functional deleterious effects calculated by SIFT and PolyPhen (Table S8), although functional studies on these variants are lacking. Impaired erythropoiesis in PRCA might arise from an altered methylation status derived from *TET2* mutations of either germline or somatic changes.

Somatic *POT1* variants have been identified in lymphoid neoplasms, such as chronic lymphocytic leukemia [35] and NK/T cell lymphoma [36]. Germline *POT1* variants are associated with clonal hematopoiesis, T cell clonality, and various solid and hematological malignancies [37]. Whether or not telomere length and telomerase activity are affected in PRCA in relation to abnormal cellular immunity or impaired erythropoiesis is unclear; however, *POT1*-mutated PRCA might be a subtype with certain clinicopathological characteristics.

PRCA and AA share several clinical features among acquired bone marrow failure syndromes, including T-cell abnormalities, efficacy of immunosuppressive treatments, and decreased hematopoietic progenitor cells. However, the mutational profiles of these two diseases were considerably different in our study and previous reports, which suggests unique features of PRCA among bone marrow failure syndromes [16–18, 38].

In conclusion, variants of CH-related genes and other genes, such as *STAT3* and *POT1*, were recurrently found in patients with PRCA, and mutations in these genes may play important roles in the pathophysiology of PRCA.

## Supplementary Information

Below is the link to the electronic supplementary material.

Supplementary file1 (DOCX 975 KB)

Supplementary file2 (XLSX 30.1 KB)

Supplementary file3 (XLSX 28.5 KB)

Supplementary file4 (XLSX 14.1 KB)

## Data Availability

Sequencing data were deposited in the Japanese Genotype–Phenotype Archive (JGA) under Accession Code JGAS000658 and JGAS000709.

## References

[1] Means RT Jr (2016) Pure red cell aplasia. Blood 128(21):2504–2509. 10.1182/blood-2016-05-717140 27881371 10.1182/blood-2016-05-717140

[2] Hara TMY, Nagata M, Okabe Y, Taniguchi S, Harada M, Niho Y, Oshimi K, Ohga S, Yoshikai Y, Nomoto K, Yumura K, Kawa-Ha F, Ueda K (1990) Human gamma delta T-cell receptor-positive cell-mediated inhibition of erythropoiesis in vitro in a patient with type I autoimmune polyglandular syndrome and pure red blood cell aplasia. Blood 75(4):941–950 2105751

[3] Nagasawa T, Abe T, Nakagawa T (1981) Pure red cell aplasia and hypogammaglobulinemia associated with Tr-cell chronic lymphocytic leukemia. Blood 57(6):1025–1031. 10.1182/blood.V57.6.1025.1025 6971663

[4] Mangan KF, Chikkappa G, Farley PC (1982) T gamma (T gamma) cells suppress growth of erythroid colony-forming units in vitro in the pure red cell aplasia of B-cell chronic lymphocytic leukemia. J Clin Invest 70(6):1148–1156. 10.1172/jci110713 6816810 10.1172/JCI110713 PMC370331

[5] Handgretinger R, Geiselhart A, Moris A, Grau R, Teuffel O, Bethge W, Kanz L, Fisch P (1999) Pure red-cell aplasia associated with clonal expansion of granular lymphocytes expressing killer-cell inhibitory receptors. N Engl J Med 340(4):278–284. 10.1056/NEJM199901283400405 9920952 10.1056/NEJM199901283400405

[6] Lacy M, Kurtin P, Tefferi A (1996) Pure red cell aplasia: association with large granular lymphocyte leukemia and the prognostic value of cytogenetic abnormalities. Blood 87(7):3000–3006 8639922

[7] Kwong YLWK (1998) Association of pure red cell aplasia with T large granular lymphocyte leukaemia. J Clin Pathol 51(9):672–675 9930071 10.1136/jcp.51.9.672 PMC500904

[8] Masuda M, Arai Y, Okamura T, Mizoguchi H (1997) Pure red cell aplasia with thymoma: Evidence of T-cell clonal disorder. Am J Hematol 54(4):324–328. 10.1002/(sici)1096-8652(199704)54:4%3c324::Aid-ajh12%3e3.0.Co;2-b 9092690 10.1002/(sici)1096-8652(199704)54:4<324::aid-ajh12>3.0.co;2-b

[9] Masuda M, Saitoh H, Mizoguchi H (1999) Clonality of acquired primary pure red cell aplasia. Am J Hematol 62(3):193–195 10539887 10.1002/(sici)1096-8652(199911)62:3<193::aid-ajh10>3.0.co;2-d

[10] Chen Z, Chen M, Yang C, Han B (2020) Immunosuppression therapy is effective for both acquired tumor-associated and primary pure red cell aplasia: a match pair case-control study. Ann Hematol 99(7):1485–1491. 10.1007/s00277-020-04105-3 32488602 10.1007/s00277-020-04105-3

[11] Liu X, Lu X, Chen L, Yang Y, Wu X, Lu R, Wang S, Zhang J, Hong M, Zhu Y, He G, Li J (2020) Immunosuppressive therapy for elderly-acquired pure red cell aplasia: cyclosporine A may be more effective. Ann Hematol 99(3):443–449. 10.1007/s00277-020-03926-6 31970447 10.1007/s00277-020-03926-6

[12] Wu X, Wang S, Lu X, Shen W, Qiao C, Wu Y, Lu R, Wang S, Zhang J, Hong M, Zhu Y, Li J, He G (2018) Response to cyclosporine A and corticosteroids in adult patients with acquired pure red cell aplasia: serial experience at a single center. Int J Hematol 108(2):123–129. 10.1007/s12185-018-2446-y 29589280 10.1007/s12185-018-2446-y

[13] Hirokawa M, Sawada K, Fujishima N, Teramura M, Bessho M, Dan K, Tsurumi H, Nakao S, Urabe A, Fujisawa S, Yonemura Y, Kawano F, Oshimi K, Sugimoto K, Matsuda A, Karasawa M, Arai A, Komatsu N, Harigae H, Omine M, Ozawa K, Kurokawa M, Group PCS (2015) Long-term outcome of patients with acquired chronic pure red cell aplasia (PRCA) following immunosuppressive therapy: a final report of the nationwide cohort study in 2004/2006 by the Japan PRCA collaborative study group. Br J Haematol 169(6):879–886. 10.1111/bjh.13376 25807974 10.1111/bjh.13376

[14] Kawakami T, Sekiguchi N, Kobayashi J, Imi T, Matsuda K, Yamane T, Nishina S, Senoo Y, Sakai H, Ito T, Koizumi T, Hirokawa M, Nakao S, Nakazawa H, Ishida F (2018) Frequent STAT3 mutations in CD8(+) T cells from patients with pure red cell aplasia. Blood Adv 2(20):2704–2712. 10.1182/bloodadvances.2018022723 30337298 10.1182/bloodadvances.2018022723 PMC6199660

[15] Kawakami F, Kawakami T, Yamane T, Maruyama M, Kobayashi J, Nishina S, Sakai H, Higuchi Y, Hamanaka K, Hirokawa M, Nakao S, Nakazawa H, Ishida F (2022) T cell clonal expansion and STAT3 mutations: a characteristic feature of acquired chronic T cell-mediated pure red cell aplasia. Int J Hematol. 10.1007/s12185-022-03310-2 35275353 10.1007/s12185-022-03310-2

[16] Balasubramanian SK, Sadaps M, Thota S, Aly M, Przychodzen BP, Hirsch CM, Visconte V, Radivoyevitch T, Maciejewski JP (2018) Rational management approach to pure red cell aplasia. Haematologica 103(2):221–230. 10.3324/haematol.2017.175810 29217782 10.3324/haematol.2017.175810 PMC5792266

[17] Long Z, Li H, Du Y, Chen M, Zhuang J, Han B (2020) Gene mutation profile in patients with acquired pure red cell aplasia. Ann Hematol 99(8):1749–1754. 10.1007/s00277-020-04154-8 32594217 10.1007/s00277-020-04154-8

[18] Fujishima N, Kohmaru J, Koyota S, Kuba K, Saga T, Omokawa A, Moritoki Y, Ueki S, Ishida F, Nakao S, Matsuda A, Ohta A, Tohyama K, Yamasaki H, Usuki K, Nakashima Y, Sato S, Miyazaki Y, Nannya Y, Ogawa S, Sawada K, Mitani K, Hirokawa M (2021) Clonal hematopoiesis in adult pure red cell aplasia. Sci Rep 11(1):2253. 10.1038/s41598-021-81890-5 33500526 10.1038/s41598-021-81890-5 PMC7838416

[19] Hirokawa M (2015) Diagnosis and management of pure red cell aplasia. Rinsho Ketsueki 56(10):1922–1931 26458430 10.11406/rinketsu.56.1922

[20] Killick SB, Bown N, Cavenagh J, Dokal I, Foukaneli T, Hill A, Hillmen P, Ireland R, Kulasekararaj A, Mufti G, Snowden JA, Samarasinghe S, Wood A, Marsh JC, British Society for Standards in H (2016) Guidelines for the diagnosis and management of adult aplastic anaemia. Br J Haematol 172(2):187–207. 10.1111/bjh.138533 26568159 10.1111/bjh.13853

[21] Casadevall N, Cournoyer D, Marsh J, Messner H, Pallister C, Parker-Williams J, Rossert J (2004) Recommendations on haematological criteria for the diagnosis of epoetin-induced pure red cell aplasia. Eur J Haematol 73(6):389–396 15522059 10.1111/j.1600-0609.2004.00348.x

[22] Lamy T, Loughran TP Jr (2011) How I treat LGL leukemia. Blood 117(10):2764–2774. 10.1182/blood-2010-07-296962 21190991 10.1182/blood-2010-07-296962 PMC3062292

[23] Hirokawa M, Sawada K, Fujishima N, Nakao S, Urabe A, Dan K, Fujisawa S, Yonemura Y, Kawano F, Omine M, Ozawa K, Group PCS (2008) Long-term response and outcome following immunosuppressive therapy in thymoma-associated pure red cell aplasia: a nationwide cohort study in Japan by the PRCA collaborative study group. Haematologica 93(1):27–33. 10.3324/haematol.11655 18166782 10.3324/haematol.11655

[24] Niroula A, Sekar A, Murakami MA, Trinder M, Agrawal M, Wong WJ, Bick AG, Uddin MM, Gibson CJ, Griffin GK, Honigberg MC, Zekavat SM, Paruchuri K, Natarajan P, Ebert BL (2021) Distinction of lymphoid and myeloid clonal hematopoiesis. Nat Med 27(11):1921–1927. 10.1038/s41591-021-01521-4 34663986 10.1038/s41591-021-01521-4 PMC8621497

[25] Wu X, Deng J, Zhang N, Liu X, Zheng X, Yan T, Ye W, Gong Y (2022) Pedigree investigation, clinical characteristics, and prognosis analysis of haematological disease patients with germline TET2 mutation. BMC Cancer 22(1):262. 10.1186/s12885-022-09347-0 35279121 10.1186/s12885-022-09347-0 PMC8917718

[26] Duployez N, Goursaud L, Fenwarth L, Bories C, Marceau-Renaut A, Boyer T, Fournier E, Nibourel O, Roche-Lestienne C, Huet G, Beauvais D, Berthon C, Cambier N, Quesnel B, Preudhomme C (2020) Familial myeloid malignancies with germline TET2 mutation. Leukemia 34(5):1450–1453. 10.1038/s41375-019-0675-6 31827242 10.1038/s41375-019-0675-6

[27] Schaub FX, Looser R, Li S, Hao-Shen H, Lehmann T, Tichelli A, Skoda RC (2010) Clonal analysis of TET2 and JAK2 mutations suggests that TET2 can be a late event in the progression of myeloproliferative neoplasms. Blood 115(10):2003–2007. 10.1182/blood-2009-09-245381 20061559 10.1182/blood-2009-09-245381

[28] Ishida F, Matsuda K, Sekiguchi N, Makishima H, Taira C, Momose K, Nishina S, Senoo N, Sakai H, Ito T, Kwong YL (2014) STAT3 gene mutations and their association with pure red cell aplasia in large granular lymphocyte leukemia. Cancer Sci 105(3):342–346. 10.1111/cas.12341 24350896 10.1111/cas.12341 PMC4317942

[29] Moffitt AB, Dave SS (2017) Clinical applications of the genomic landscape of aggressive non-hodgkin lymphoma. J Clin Oncol 35(9):955–962. 10.1200/JCO.2016.71.7603 28297626 10.1200/JCO.2016.71.7603

[30] Cheon H, Xing JC, Moosic KB, Ung J, Chan VW, Chung DS, Toro MF, Elghawy O, Wang JS, Hamele CE, Hardison RC, Olson TL, Tan SF, Feith DJ, Ratan A, Loughran TP (2022) Genomic landscape of TCRalphabeta and TCRgammadelta T-large granular lymphocyte leukemia. Blood 139(20):3058–3072. 10.1182/blood.2021013164 35015834 10.1182/blood.2021013164 PMC9121841

[31] Belizaire R, Wong WJ, Robinette ML, Ebert BL (2023) Clonal haematopoiesis and dysregulation of the immune system. Nat Rev Immunol 23(9):595–610. 10.1038/s41577-023-00843-3 36941354 10.1038/s41577-023-00843-3 PMC11140722

[32] Park S, Yun J, Choi SY, Jeong D, Gu JY, Lee JS, Seong MW, Chang YH, Yun H, Kim HK (2023) Distinct mutational pattern of T-cell large granular lymphocyte leukemia combined with pure red cell aplasia: low mutational burden of STAT3. Sci Rep 13(1):7280. 10.1038/s41598-023-33928-z 37142644 10.1038/s41598-023-33928-z PMC10160083

[33] Yoshizato T, Dumitriu B, Hosokawa K, Makishima H, Yoshida K, Townsley D, Sato-Otsubo A, Sato Y, Liu D, Suzuki H, Wu CO, Shiraishi Y, Clemente MJ, Kataoka K, Shiozawa Y, Okuno Y, Chiba K, Tanaka H, Nagata Y, Katagiri T, Kon A, Sanada M, Scheinberg P, Miyano S, Maciejewski JP, Nakao S, Young NS, Ogawa S (2015) Somatic mutations and clonal hematopoiesis in aplastic anemia. N Engl J Med 373(1):35–47. 10.1056/NEJMoa1414799 26132940 10.1056/NEJMoa1414799 PMC7478337

[34] Jaiswal S, Fontanillas P, Flannick J, Manning A, Grauman PV, Mar BG, Lindsley RC, Mermel CH, Burtt N, Chavez A, Higgins JM, Moltchanov V, Kuo FC, Kluk MJ, Henderson B, Kinnunen L, Koistinen HA, Ladenvall C, Getz G, Correa A, Banahan BF, Gabriel S, Kathiresan S, Stringham HM, McCarthy MI, Boehnke M, Tuomilehto J, Haiman C, Groop L, Atzmon G, Wilson JG, Neuberg D, Altshuler D, Ebert BL (2014) Age-related clonal hematopoiesis associated with adverse outcomes. N Engl J Med 371(26):2488–2498. 10.1056/NEJMoa1408617 25426837 10.1056/NEJMoa1408617 PMC4306669

[35] Mansouri L, Thorvaldsdottir B, Sutton LA, Karakatsoulis G, Meggendorfer M, Parker H, Nadeu F, Brieghel C, Laidou S, Moia R, Rossi D, Catherwood M, Kotaskova J, Delgado J, Rodriguez-Vicente AE, Benito R, Rigolin GM, Bonfiglio S, Scarfo L, Mattsson M, Davis Z, Gogia A, Rani L, Baliakas P, Foroughi-Asl H, Jylha C, Skaftason A, Rapado I, Miras F, Martinez-Lopez J, de la Serna J, Rivas JMH, Thornton P, Larrayoz MJ, Calasanz MJ, Fesus V, Matrai Z, Bodor C, Smedby KE, Espinet B, Puiggros A, Gupta R, Bullinger L, Bosch F, Tazon-Vega B, Baran-Marszak F, Oscier D, Nguyen-Khac F, Zenz T, Terol MJ, Cuneo A, Hernandez-Sanchez M, Pospisilova S, Mills K, Gaidano G, Niemann CU, Campo E, Strefford JC, Ghia P, Stamatopoulos K, Rosenquist R (2023) Different prognostic impact of recurrent gene mutations in chronic lymphocytic leukemia depending on IGHV gene somatic hypermutation status: a study by ERIC in HARMONY. Leukemia 37(2):339–347. 10.1038/s41375-022-01802-y 36566271 10.1038/s41375-022-01802-y PMC9898037

[36] Li S, Liu T, Liu H, Zhai X, Cao T, Yu H, Hong W, Lin X, Li M, Huang Y, Xiao J (2022) Integrated driver mutations profile of chinese gastrointestinal-natural killer/T-cell lymphoma. Front Oncol 12:976762. 10.3389/fonc.2022.976762 36059700 10.3389/fonc.2022.976762 PMC9434212

[37] DeBoy EA, Tassia MG, Schratz KE, Yan SM, Cosner ZL, McNally EJ, Gable DL, Xiang Z, Lombard DB, Antonarakis ES, Gocke CD, McCoy RC, Armanios M (2023) Familial clonal hematopoiesis in a long telomere syndrome. N Engl J Med 388(26):2422–2433. 10.1056/NEJMoa2300503 37140166 10.1056/NEJMoa2300503 PMC10501156

[38] Zhang X, Shi Y, Song L, Shen C, Cai Q, Zhang Z, Wu J, Fu G, Shen W (2018) Identification of mutations in patients with acquired pure red cell aplasia. Acta Biochim Biophys Sin (Shanghai) 50(7):685–692. 10.1093/abbs/gmy052 29767669 10.1093/abbs/gmy052

