# Supplementary material for: Mutational heterogeneities in STAT3 and clonal hematopoiesis-related genes in acquired pure red cell aplasia
Source: Ann Hematol. 2025 Apr 9;104(3):1471–9. doi: 10.1007/s00277-025-06356-4 (PMC12031804; doi:10.1007/s00277-025-06356-4)
Supplement: Supplementary file 1 — Supplementary file1 (DOCX 975 KB) [file 277_2025_6356_MOESM1_ESM.docx]

**Supplemental Materials**

Supplement to: Kawakami T, et al. **Mutational heterogeneities in patients with acquired pure red cell aplasia**

**Contents:**

Supplemental Methods page 2

Supplemental Figures

Figure S1 page 4

Figure S2 page 5

Figure S3 page 6

Figure S4 page 7

Figure S5 page 8

Figure S6 page 9

Figure S7 page 10

Figure S8 page 11

Supplemental Tables

Table S1 page 12

Table S2 page 13

Table S3, 4 page 14

Table S5 page 15

Table S6 page 16

Table S7 page 17

Table S8 page 18

Table S9 page 19

Table S10 page 20

Reference page 21

**Supplemental Methods**

*DNA extraction*

Mononuclear cells (MNCs) were isolated from peripheral blood or bone marrow using Ficoll gradient separation (GE Healthcare, Little Chalfont, Buckinghamshire, UK) and stored at -80 °C until DNA extraction. DNA was extracted using the QIAamp DNA Blood Mini Kit (QIAGEN GmbH, Hilden, Germany) according to the manufacturer’s instructions.

*Sorting of cell subpopulations*

In select patients, target cell subpopulations were separated using fluorescence-activated cell sorter (FACS). For example, CD3^+^CD4^-^CD8^+^ T (CD8^+^ T) cells or CD3^+^CD4^+^CD8^-^ T (CD4^+^ T) cells were separated using antibodies against CD3 (APC, clone SP34-2; BD Biosciences, Franklin Lakes, NJ, USA), CD4 (PerCP, clone L200; BD Biosciences), and CD8 (PE, clone RPA-T8; BD Biosciences) using a FACSAria cell sorter (BD Biosciences) (Figure S8).

*Validation of candidate somatic variants by Sanger sequencing*

PCR amplification was performed using primers for Sanger sequencing (Table S10), and the PCR products were purified by gel electrophoresis, followed by extraction with a QIAExII Gel Extraction kit (QIAGEN) or Agencourt AMPure XP beads (Beckman Coulter, Brea, CA, USA). The purified PCR products were then sequenced using a BigDye v1.1 Cycle Sequencing kit and an ABI Prism 3500 Genetic Analyzer (Thermo Fisher Scientific).

*Statistical analyses*

Comparisons between different groups were performed using Fisher’s exact test, two-sided *t*-test, Mann-Whitney U test, or Kruskal-Wallis’s test, as appropriate. Steel-Dwass test and Bonferroni correction were applied as post hoc analyses for Kruskal-Wallis’s test and Fisher’s exast test, respectively. *p*-values of <0.05 was considered to indicate statistical significance. All statistical analyses were performed using EZR software (ver. 1.55)^1^.

**Figure S1**

**Variants detected in whole-exome sequencing.** Each column represents individual patients. Genes are compiled by their representative function. PRCA, pure red cell aplasia.

**Figure S2**


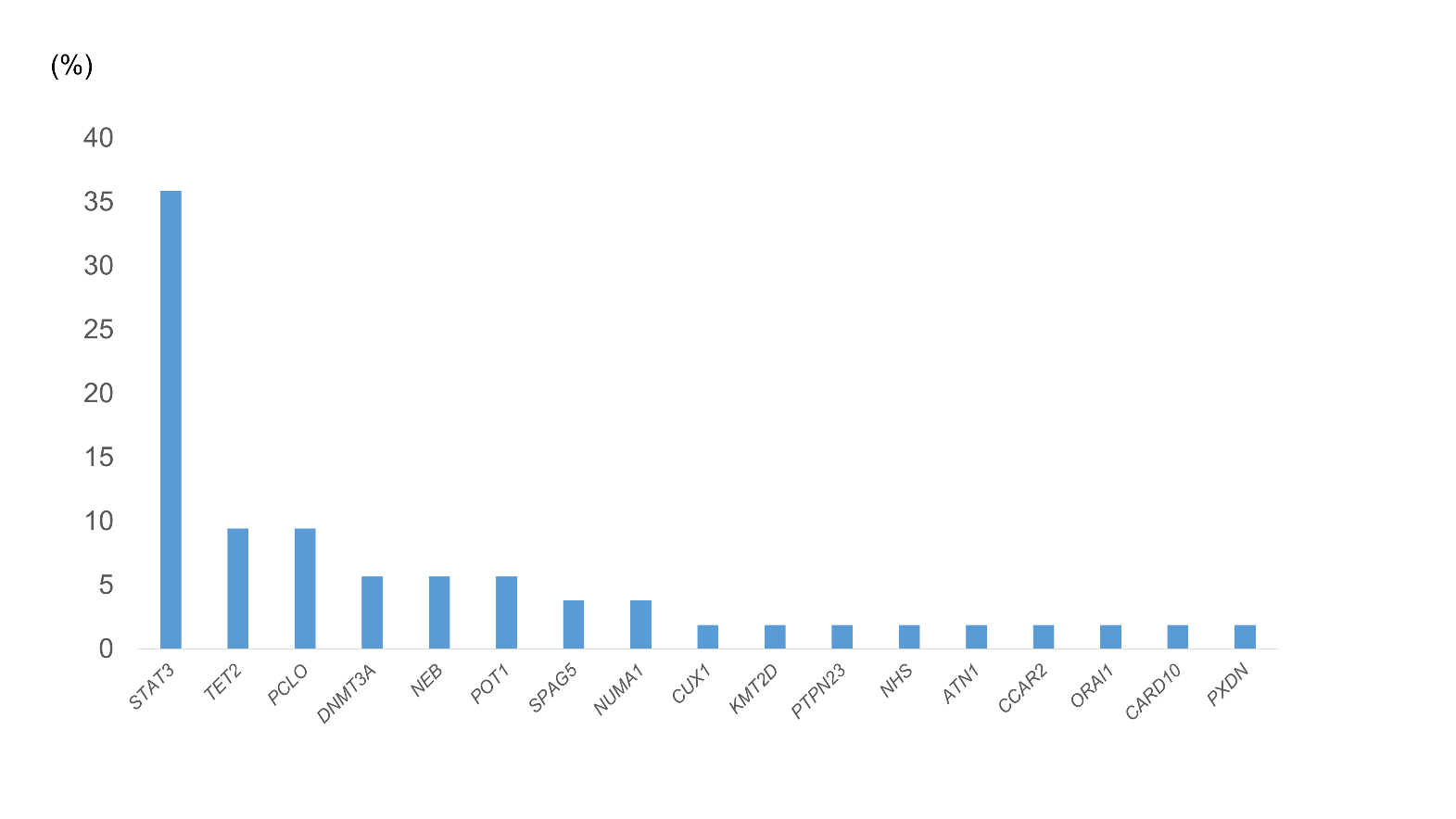


**Frequently mutated genes in PRCA.** This bar graph shows the frequencies of gene mutations in 53 PRCA patients. PRCA, pure red cell aplasia

**Figure S3**


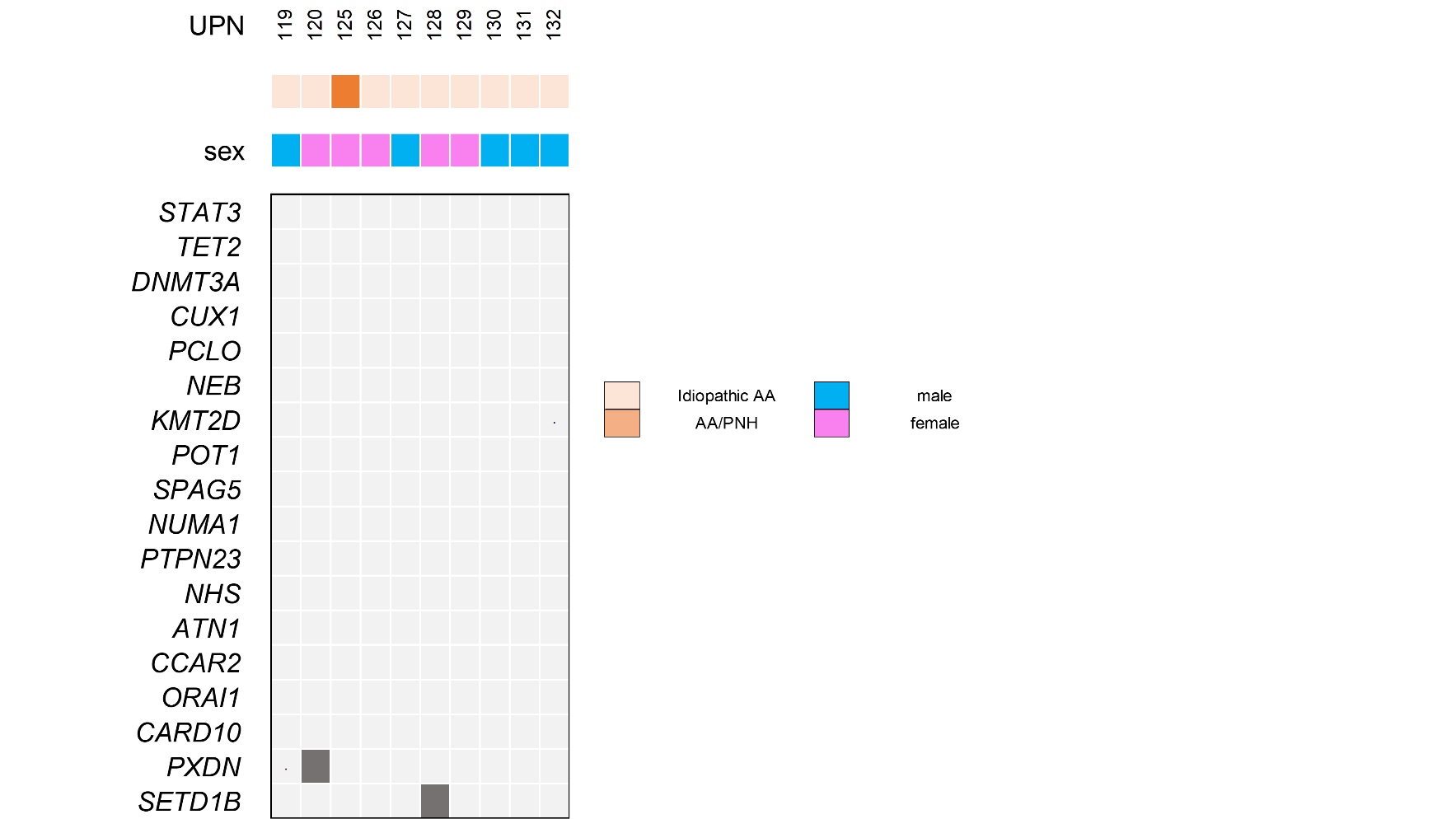


**Genes mutational profile of AA patients.** The columns indicate individual patients and healthy controls. UPN, unique patient number; AA, aplastic anemia; PNH, paroxysmal nocturnal hemoglobinuria.

**Figure S4**


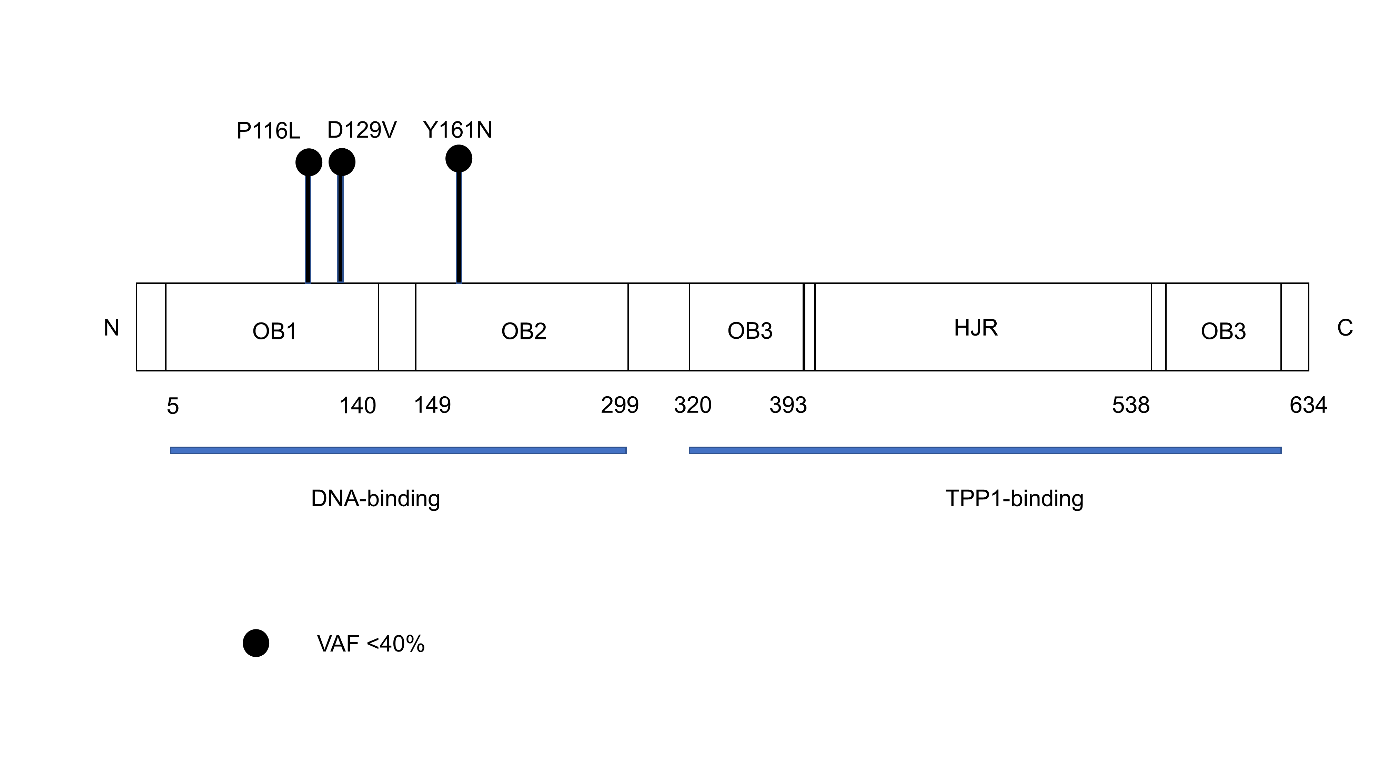


**The distribution of *POT1* variants.** Each circle represents variants. OB, oligonucleotide-binding; HJR, holiday junction resolvase-like

**Figure S5**

**
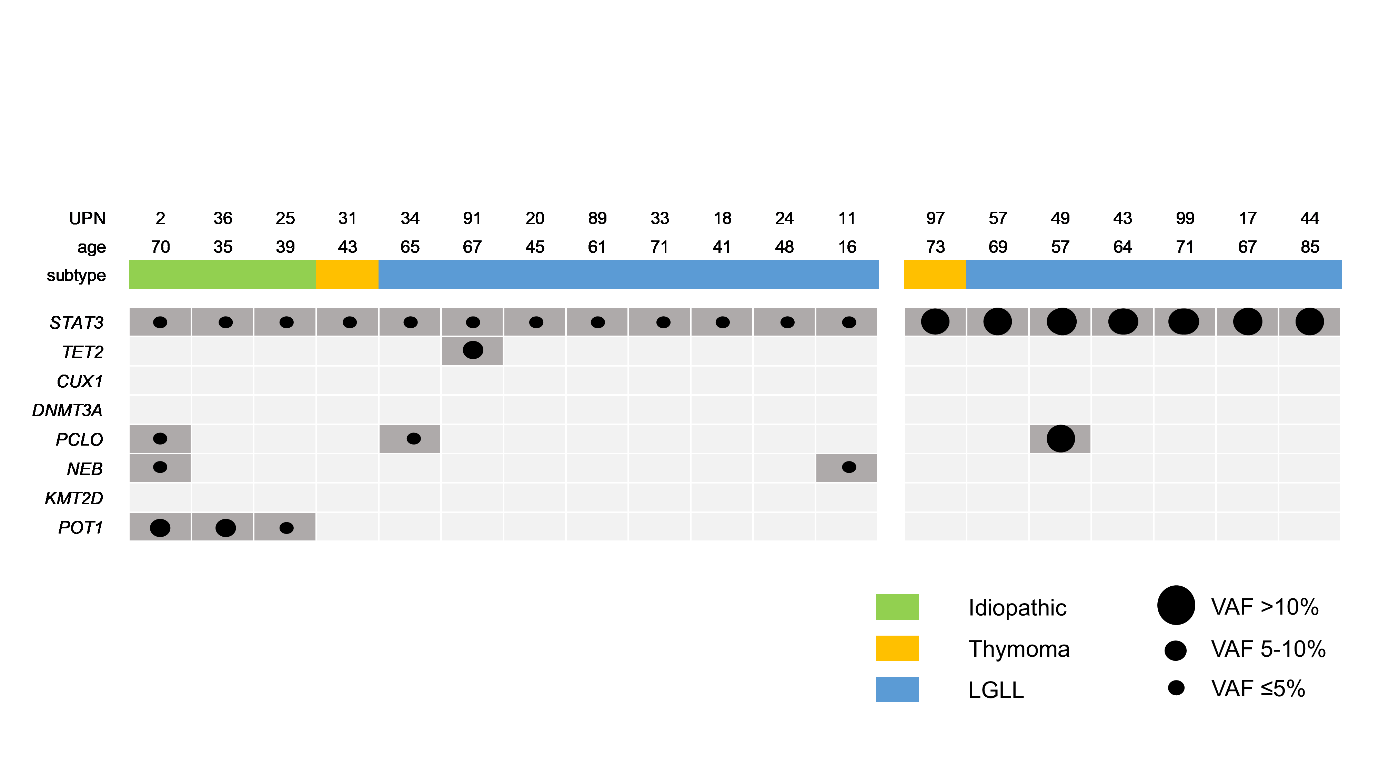
**

**Variant allele frequencies (VAFs) of *STAT3*, clonal hematopoiesis-related genes and *POT1* variants.** Each row represents a gene, and the column~~s~~ represent an individual patient. Figure shown in each circle corresponds to value of VAFs. The backgrounds of PRCA are color-coded at the top. UPN, unique patient number; LGLL, large granular lymphocytic leukemia.

**Figure S6**


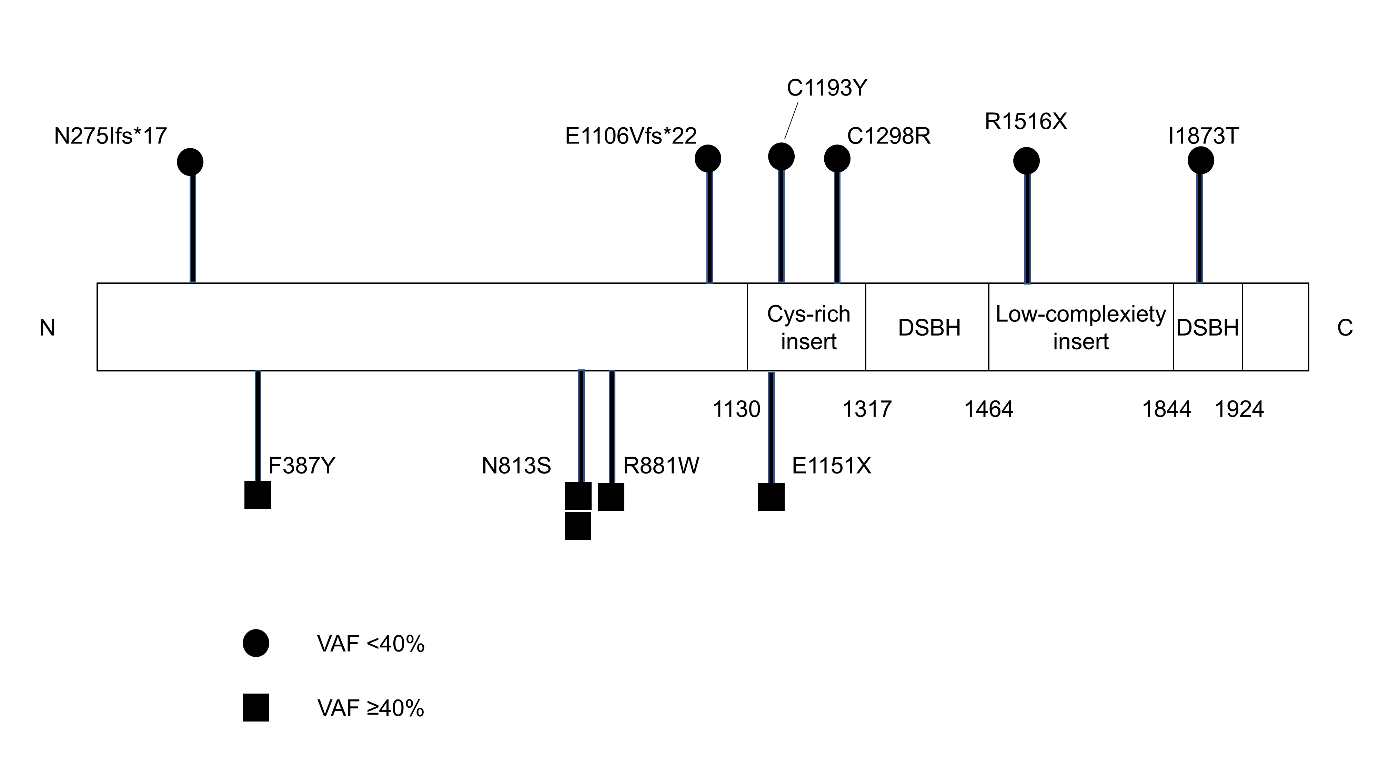


**The distribution of *TET2* variants.** Each circle and square represents variants. DSBH, double-stranded b helix

**Figure S7**

**
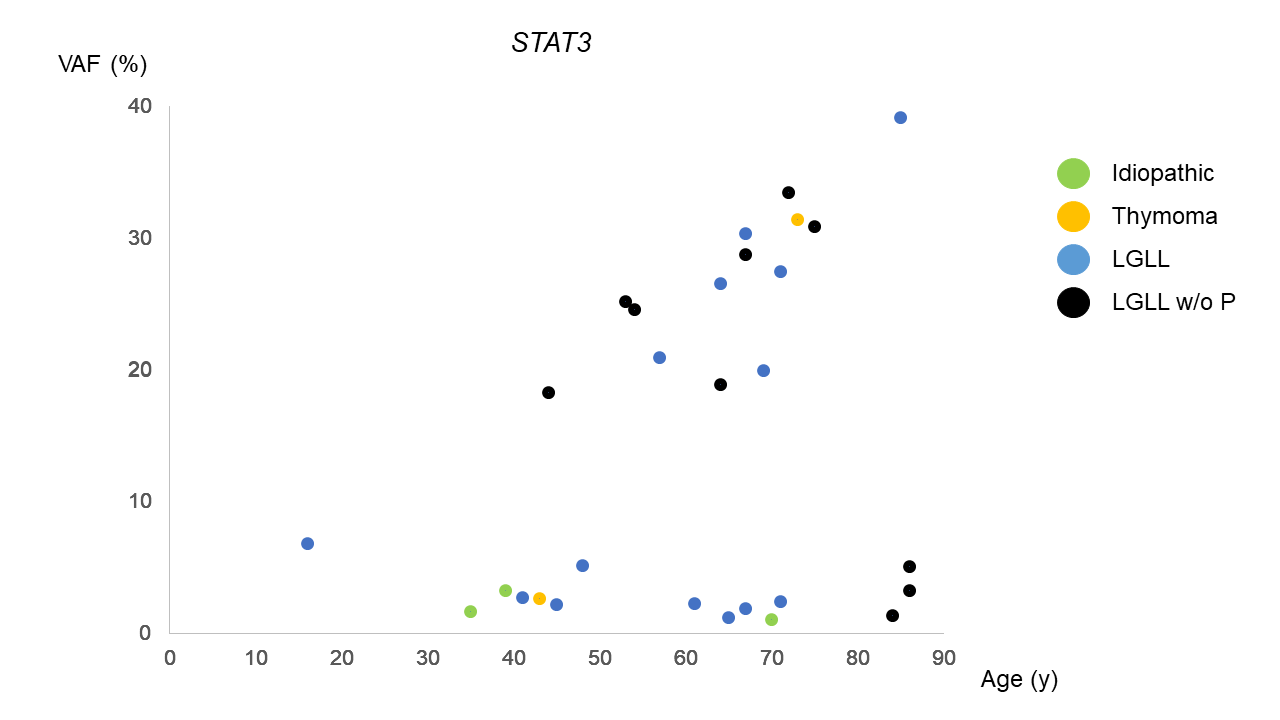
**

**Age of patients and variant allele frequencies (VAFs) of *STAT3* variants in pure red cell aplasia.** Each dot represents one patient. LGLL, large granular lymphocytic leukemia; w/o P, without pure ed cell aplasia.

**Figure S8**

**
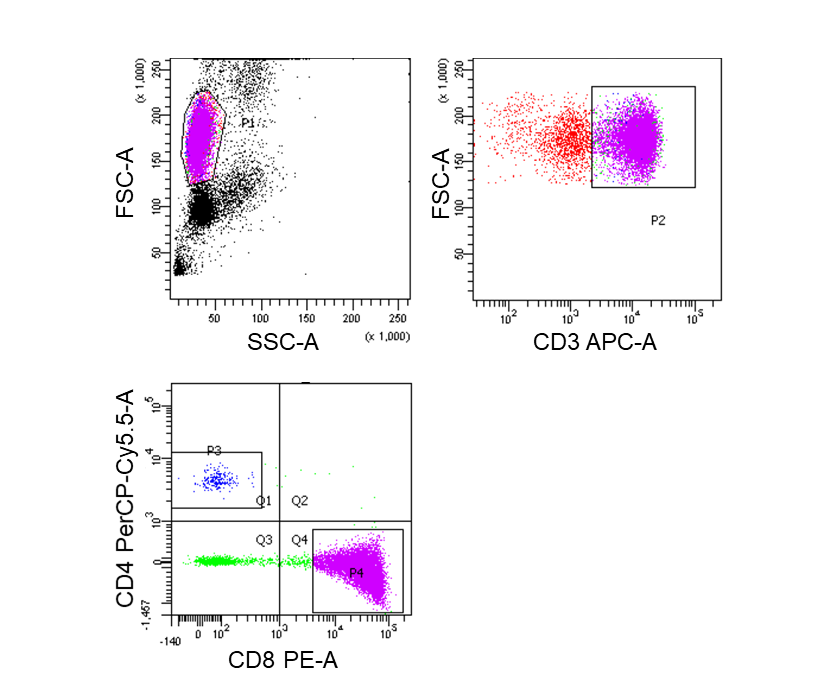
**

**Representative examples of scatterplots of sorted lymphocytes.** CD3^+^8^+^ T cells and CD3^+^CD4^+^ T cells were separated for whole-exome sequencing. FSC-A, forward-scatter area; SSC-A, side-scatter area; APC-A, allophycocyanin area; PerCP-Cy5.5-A, peridinin chlorophyll protein-cyanin5.5 area; PE-A, phycoerythrin area.

**Table S1: Diagnostic criteria**

BM, bone marrow; MRI, magnetic resonance imaging

**Table S2: Genes analyzed in targeted sequencing**

**Table S3: Results of whole-exome sequencing**.

**Table S4: Results of targeted sequencing**

These tables are shown in a separate file.

**Table S5: Comparison of PRCA patients with and without *STAT3* variants**

*Significantly different

PRCA, pure red cell aplasia; CsA, cyclosporine; CY, cyclophosphamide

**Table S6: Comparison based on the number of mutated genes**

*Significantly different

PRCA, pure red cell aplasia; CsA, cyclosporine; CY, cyclophosphamide

**Table S7: Summary of *POT1* variants detected in this study.**

UPN, unique patient number; PRCA, pure red cell aplasia; CsA, cyclosporin A; mPSL, methyl prednisolone; PSL, prednisolone; CY, cyclophosphamide; PR, partial response; complete response; NR, no response

**Table S8: Summary of *TET2* variants detected in this study**

UPN, unique patient number; PRCA, pure red cell aplasia; VAF, variant allele frequency; SLE, systemic lupus erythematosus; AIH, autoimmune hepatitis; LGLL, large granular lymphocytic leukemia; AIP, autoimmune pancreatitis.

**Table S9: Results of targeted sequencing in LGLL patients without PRCA**.

This table is shown in a separate file.

**Table S10: Primers for Sanger sequencing**

**REFERENCE**

1. Kanda Y. Investigation of the freely available easy-to-use software 'EZR' for medical statistics. Bone Marrow Transplant. 2013;48(3):452-458.
